# Lens Antenna Arrays for THz Superconducting HEB Mixers: A Review and a Metasurface Coupling Approach

**DOI:** 10.3390/s26103258

**Published:** 2026-05-21

**Authors:** Yuner Gan, Ruiguang Peng, Shijia Feng, Maimai Mu, Qian Wang

**Affiliations:** 1 Institute of New Materials and Advanced Manufacturing, Beijing Academy of Science and Technology, Beijing 100094, China; pengruiguang@bjast.ac.cn (R.P.); fengshijia@bjast.ac.cn (S.F.); 2 National Astronomical Observatories, Chinese Academy of Sciences, Beijing 100101, China; mumm@bao.ac.cn; 3 Low Temperature Detector Laboratory, Department of Astronomy, Tsinghua University, Beijing 100084, China

**Keywords:** terahertz, hot electron bolometer (HEB), planar antenna, quasi-optical coupling, metasurface, beam shaping

## Abstract

Terahertz hot electron bolometer (HEB) mixers, which offer the highest sensitivity in the frequency range above 1.5 THz, are equipped on space observatories to detect the terahertz radiation emitted from the interstellar medium within galaxies. To increase the mapping speed, it is essential to develop large HEB mixer arrays. However, conventional quasi-optical coupling methods, including single large silicon lens approaches and silicon lens array approaches, suffer from the conflict of achieving high filling factor and uniform illumination on the HEB mixer array. This paper reviews the research progress on quasi-optical coupled HEB mixer arrays and proposes an innovative array coupling scheme to overcome the existing limitation. We designed a metasurface beam shaper based on the Gerchberg–Saxton algorithm and COMSOL simulation to transform an incoming Gaussian beam into a flattop beam in the focal plane, thereby forming uniform illumination for an antenna-coupled HEB mixer array. The metasurface is intended primarily for uniform local oscillator (LO) distribution across the array. The simulation of the metasurface beam shaper at 0.6 THz demonstrates a flattop beam with a flat region approximately 3 mm wide, and the intensity across this region varies by only 4.2%. The same simulation is also performed at 1.6 THz, and the flat region is 1.5 mm wide with a 5.5% intensity variation. This work demonstrates the feasibility of using a metasurface to convert a Gaussian beam into a flattop beam at terahertz frequencies as well as a pathway for array-level coupling schemes for HEB mixer arrays with high filling factor and uniform illumination.

## 1. Introduction

Situated between microwave and infrared wave, the terahertz (THz) frequency band (0.1–10 THz) has great potential in a wide range of applications, including high-resolution molecular and atomic spectroscopy for atmospheric and astronomical science, high-speed communication, and non-destructive imaging for security and medical diagnostics [1]. Different from direction detection, which detects and amplifies the signal directly, heterodyne detection uses a mixer to mix a local oscillator (LO) signal and the detected signal, converting the radiation from the THz region to the gigahertz (GHz) region with a high spectrum resolution $∆R\geq 10^{7}$ [2]. Thus, heterodyne receivers are used in astronomical observatories to detect the molecular rotational lines and atomic fine structure lines at THz frequencies emitted from the interstellar medium (ISM) within galaxies, thus tracking the life cycle of the ISM and thus enabling an understanding of the process of galaxy evolution and star formation [3,4,5].

Superconductor–Insulator–Superconductor (SIS) mixers, which offer quantum-limited sensitivity, have been demonstrated in NbN-based devices with robust performance at ~650 GHz [6,7,8]. Above ~1.5 THz, however, hot electron bolometer (HEB) mixers based on niobium nitride (NbN) superconducting thin films become the detector of choice. Operating at 4.2 K, they offer receiver noise temperatures below 10 times the quantum limit and relatively wide intermediate frequency (IF) bandwidths of ~3 GHz [9,10,11]. Their excellent sensitivity and stability make them the detector of choice for airborne and space-borne observatories, such as the Herschel Space Observatory [12], the Stratospheric Observatory for Infrared Astronomy (SOFIA) [13], and the Galactic/Extragalactic ULDB Spectroscopic Terahertz Observatory (GUSTO) [14]. Recently, the HEB mixers based on magnesium diboride (MgB_2_) thin films have been reported to have low receiver noise temperatures approaching 10 times the quantum limit, IF bandwidths 3–4 times larger than NbN HEB mixers, and higher working temperatures (>20 K) [15]. The latter characteristic reduces the cooling system cost by at least 60%, making the MgB_2_ HEB mixer a promising replacement for the NbN HEB in the future.

HEB mixers could be divided into two types depending on the optical coupling method: waveguide-coupled HEB mixers [16] and quasi-optically coupled HEB mixers [17]. In the supra-THz band (>1 THz), the structure of the waveguides becomes more delicate, and their minimum size becomes so small that it is difficult to fabricate. Therefore, the quasi-optical coupling approach is a better choice for the HEB mixer at supra-THz, which integrates a planar antenna with the HEB bridge in a substrate that is placed in the focal plane of an optical element, either an elliptical/hyper-hemisphere silicon lens, a metasurface lens, or a metallic parabolic mirror. Various planar antennas have been successfully integrated with HEBs, including broadband self-complementary antennas like the spiral and log-periodic antenna, and narrowband resonant antennas such as the twin-slot and annular-slot antenna.

Due to the limitation on the cooling power and LO power, the pixel number of the current HEB mixer array is below 20. However, to map a larger area of the sky in the limited lifetime of a space observatory, the development of a large heterodyne receiver array is necessary. The transition from a single pixel to an array faces multiple challenges, with the design of quasi-optical HEB mixer arrays being particularly critical. Considerations need to be taken into account in the array architecture, the design and fabrication of the optical element, the planar-antenna type, minimization of crosstalk between pixels, the uniformity of performance across the array, and the overall integration complexity.

This paper presents a review of quasi-optical HEB mixer arrays, with a focus on planar-antenna-coupled configurations. We first introduce the configuration of a single-pixel quasi-optically coupled HEB mixer, survey different types of optical elements and planar antennas, and discuss the achieved performance of HEB mixers that employ these designs. Then we summarize different kinds of planar-antenna HEB mixer arrays and their reported performance metrics, such as noise temperature and crosstalk, and highlight their integration into astronomical instruments. Finally, we propose and simulate a metasurface-based coupling scheme to achieve both a high filling factor and uniform illumination. We design a metasurface beam shaper to transform a Gaussian beam into a flattop beam and evaluate its free-space performance as a potential means to enable uniform illumination for future HEB arrays.

## 2. Quasi-Optically Coupled HEB Mixers

### 2.1. Configuration of Quasi-Optical HEB Mixers

**Figure 1:**
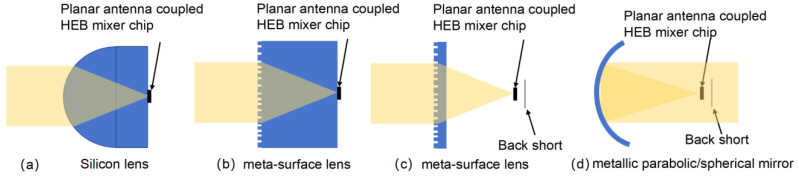
(**a**) Silicon-lens-coupled planar-antenna HEB mixer. The HEB chip is attached to the backside of the lens. (**b**,**c**) Metasurface-lens-coupled planar-antenna HEB mixer. For (**b**) the HEB chip is attached to the back of the lens material. (**d**) Metallic parabolic/spherical-mirror-coupled planar-antenna HEB mixer. The HEB chips in (**c**) and (**d**) are placed in the focal point of the optical element, and a back short is placed at a quarter-wavelength distance on the back of the HEB chip to reduce the radiation loss.

A quasi-optically coupled HEB mixer contains two parts: an optical element and a planar-antenna-coupled HEB mixer chip. The planar-antenna-coupled HEB mixer chip is located in the focal plane of the optical element, and the center of the HEB superconducting bridge coincides with the focal point. The optical element focuses the incoming THz and the LO radiation onto the HEB chip, where the planar antenna receives it and delivers the power to the HEB bridge. Figure 1a–d shows the HEB mixer based on a silicon lens, a metasurface lens, and a metallic parabolic/spherical mirror, respectively. For the lens scheme in Figure 1a and the metasurface lens scheme in Figure 1b, the HEB chip is attached to the back of the lens. When the incident angle exceeds the critical angle, the incident wave undergoes total reflection at the substrate air interface and is trapped by the substrate; therefore, most radiation is concentrated on the focus of the optical element, while for the other two schemes shown in Figure 1c,d, the optical element and HEB chip need to be mounted separately, and some of the radiation will pass through the HEB chip, resulting in signal loss. This loss can be greatly reduced by placing a back short at a quarter-wavelength distance on the back of the HEB chip.

### 2.2. Different Kinds of Optical Elements

The optical element arrays that were reported for coupling THz radiation into detectors have been summarized in Table 1. This includes lens arrays as well as metasurfaces. There have been reports of silicon lens arrays made via direct machining, laser ablation, and photolithography [18,19,20,21,22,23]. These arrays have sizes of up to ~1000 pixels for MKID arrays. Despite this, the applicability of these techniques to supra-THz HEB arrays poses difficulties in terms of alignment accuracy and filling factor (Section 3).

With the development of metamaterials, lens arrays based on metasurface materials have also been explored. A 5 × 5 metasurface-based lens array working at a frequency range from 0.35 to 1.05 THz was reported by Wang et al. [24]. The focusing of the THz light is achieved by phase modulation using C-shape split-ring resonators. Due to the excellent processability of silicon, a silicon-etched gradient-index (GRIN) lens array has also been demonstrated at 90 and 150 GHz by Pisano et al. [26]. However, this approach is not suitable for higher frequencies since the feature size becomes too small to fabricate. For higher frequencies, Pisano et al. developed silicon-embedded metal-mesh GRIN lenses [26], which use photolithographically patterned metal squares on silicon stacks to achieve a gradient index. Recently, Ren et al. reported a metalens based on the structure of Si micropillars on a thin Si substrate [27]. Phase modulation is achieved by the Si micropillars. They used the metalens to couple a NbN HEB mixer and obtained a noise temperature of 1800 K at 1.63 THz and a power coupling efficiency of 25% from free space to the mixer. This scheme could be applied to HEB mixer arrays.

A metallic parabolic/spherical mirror could achieve >95% reflection of THz waves; thus, it has been used to couple THz waves into HEB mixers [28,29]. S. Cherednichenko et al. used a 16-pixel collimating mirror array to couple THz radiation into a planar double-dipole-antenna-coupled HEB mixer array [25]. However, due to the difficulty in achieving precise alignment between its focal point and the center of the HEB, the parabolic mirror array is not suitable for coupling large HEB mixer arrays.

### 2.3. Different Types of Planar Antenna

Previous studies have investigated the design, fabrication, and performance of different kinds of planar antennas, including broadband bow-tie, log-spiral, and log-periodic antennas, as well as narrowband twin-slot and annular-slot antennas. Due to the simple shape and wideband input impedance of the bow-tie antenna, it has been used in various applications since the 1950s, including ground penetrating radar, Wi-Fi, wireless and microwave imaging-based applications. Gol’tsman et al. investigated the bolometric response of bow-tie-antenna-coupled YBa_2_Cu_3_O_7_-δ (YBCO) hot electron bolometers [30]. However, with the increasing working frequency, deterioration occurs in the radiation pattern of this type of antenna.

**Figure 2:**
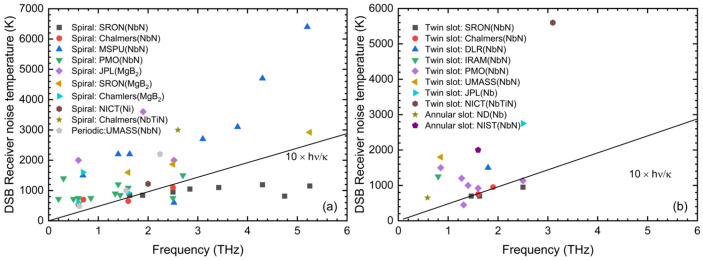
(**a**) State-of-the-art performance of planar self-complementary-antenna-coupled HEB mixers, including spiral-antenna-coupled HEB mixers and periodic-antenna-coupled HEB mixers; (**b**) state-of-the-art performance of narrowband antenna-coupled HEB mixers, including twin-slot antennas and annular antennas.

The advantages of planar self-complementary antennas, including log-spiral and log-periodic antennas, are their broadband performance, because their impedance remains nearly constant at a 10:1 frequency range, and they do not need an extra radio-frequency filter to prevent the radiation from traveling to the edge of the antenna. From the year 2000, different research groups have investigated the performance of hot electron mixers coupled with log-spiral and log-periodic antennas, as shown in Figure 2a [31,32,33,34,35,36,37,38]. Gao et al. from SRON have demonstrated spiral-antenna-coupled NbN HEB mixers working at different frequencies, from 1.4 to 5.3 THz, and the receiver noise temperature reached below 10 times quantum noise [9,10,11,17,39,40,41]. This group also reported spiral-antenna-coupled HEB mixers based on MgB_2_ thin film working at 1.6 THz, 2.5 THz, and 5.3 THz, and the receiver noise temperatures were just above 10 times quantum noise [15,42]. The spiral-antenna-coupled HEB mixers based on NbN thin film and MgB_2_ thin film, demonstrated by Cherednichenko et al. from Chalmers University of Technology, also show good performance and their receiver noise temperatures are around 10 times quantum noise [43,44,45,46]. Cai et al. from PMO fabricated spiral-antenna-coupled NbN HEB mixers and measured them with LO frequency from 0.2 to 2.7 THz, and they all showed good sensitivity [47,48,49,50,51].

Due to the high polarization purity and impedance control capabilities, narrowband antennas such as twin-slot and annular-slot antennas can efficiently harness the power of the LO source while adapting to various detectors. Therefore, the HEB mixers equipped in the ground- and space-based missions commonly use twin-slot antenna-coupled HEB mixers. Figure 2b shows the DSB receiver noise temperature of twin-slot antennas and annular-antenna-coupled HEB mixers [52,53,54,55,56,57,58,59,60,61]. A twin-slot-antenna-integrated HEB mixer with a receiver noise temperature of 1200 K at 237 μm designed for TREND (Terahertz REceiver with NbN HER Device) on the 1.7-m diameter AST/RO submillimeter-wave telescope at the Amundsen/Scott South Pole Station was reported by Gerecht et al. in 2003 [62]. In 2005, W. Jellema et al. demonstrated phase-sensitive beam pattern measurements of twin-slot HEB lens-antenna mixers for the Heterodyne Instrument for Far-Infrared (HIFI) at 1.1, 1.2, and 1.6 THz [63]. In 2008, Cherednichenko et al. investigated the performance of twin-slot-antenna-integrated NbN HEB mixers used in the HIFI instrument of the Herschel Space Observatory and obtained a noise temperature of 1100 K at 1.6 THz and a noise temperature of 1450 K at 1.9 THz [64]. In the same year, Semenov et al. demonstrated a 1.8 THz hot electron bolometer mixer for the balloon mission Terahertz and submillimeter Limb Sounder (TELIS), achieving a DSB receiver noise temperature of 1500 K [65]. In 2015, Zhou et al. reported the performance of a twin-slot-antenna-coupled NbN HEB mixer aimed for a ground-based 5 m terahertz telescope (DATE5) in DOME A and obtained a noise temperature of 600 K at 1.3 THz [66].

Although narrowband antennas exhibit excellent noise temperature in narrowband applications, their relative bandwidth essentially limits their application in broadband spectroscopy observations. Therefore, arrays based on narrowband antennas are the preferred choice in observation tasks that pursue extremely high sensitivity, while arrays based on self-complementary antennas are more suitable for applications that require wide spectral coverage.

## 3. Planar-Antenna Arrays for HEB Mixers

For quasi-optical coupled HEB arrays, there are commonly two approaches to array configuration. One is called the fly-eye approach, which consists of an array of lenses, where each lens integrates with a single HEB mixer. Another approach uses a single lens with a separate silicon wafer containing an array of HEB mixers. The main advantage of the first approach is that each detector has its own focusing element, so that better uniformity is achieved across the mixer array, while for the second approach, beam distortion exists due to the fact that only one detector is placed at the center of the lens, and other detectors are all placed off-axis relative to the lens. The main disadvantage of the first approach is that the array’s filling factor is limited by the lens diameter since the detectors cannot be spaced closer together than the diameter of the lens, while for the second approach, the array filling factor will not be limited by the lens diameter but by the array dimension, leading to a much larger filling factor [25].

Mixer arrays based on various antennas were reported by different research groups, and most of them adopted the fly-eye scheme with a silicon lens array, such as the arrays in [67], which is a 3-pixel lens-antenna-coupled mixer array concept based on either twin-slot or slot-ring antennas. The mixer array consists of two layers: the silicon lens array and the chip of the mixer array integrated with MMIC IF amplifiers. The measured DSB receiver noise temperature of the mixer in the array at 1.6 THz is around 3000 K. This group has also proposed a large-pixel mixer array concept, containing a lens array, a log-periodic-coupled mixer array chip, an MMIC IF amplifier array, and a dc bias board [68]. In 2015, Gao et al. investigated a 2 × 2-pixel NbN hot electron bolometer (HEB) array receiver using twin-slot antennas [69]. In 2022, the same group demonstrated three 4 × 2-pixel NbN HEB mixer arrays at 1.4 THz, 1.9 THz, and 4.7 THz. These arrays, flown onboard the GUSTO balloon-borne observatory, represent the present benchmark for quasi-optical supra-THz HEB mixer array performance and uniformity. They achieved DSB receiver noise temperatures of 330 K, 420 K, and 700 K at the three respective frequencies, and the pixel-to-pixel variation in coupling efficiency within an array is less than 4% at 4.7 THz in the worst case [70,71].

The fly-eye approaches have also used collimating mirrors as the coupling optical elements. For instance, S. Cherednichenko et al. designed a compact 16-pixel fly eye approach array working at 2.5 THz, which uses collimating mirrors as focusing elements, planar double dipole antennas on a membrane as optical coupling elements, and NbN HEB mixers on Si_3_N_4_/SiO_2_ membranes as the mixing element. The gain bandwidth of the mixers is measured to be 0.7–0.9 GHz, which is much smaller than that of the NbN HEB mixer silicon substrate [25].

For the single-lens approach, Liu et al. demonstrated a focal plane array based on planar annular-slot antennas coupled with niobium hot electron bolometers [60]. A 4-pixel antenna-coupled HEB mixer array was attached to a single silicon lens. At 0.585 THz, a spatial resolution of 2.75 mm was achieved, and for two adjacent elements in the mixer array, the DSB mixer noise temperatures were measured to be 1675 and 3517 K, and the mixer conversion gains were measured to be 14.73 and 17.74 dB.

For an antenna-coupled HEB mixer array, the investigation of the crosstalk on the neighboring antenna is essential. Researchers have simulated the crosstalk of an antenna array to obtain the minimum distance between neighboring antennas. J. Baubert et al. simulated the crosstalk of a 2 × 2-pixel twin-slot-antenna-coupled mixer array located in the center of a spherical mirror array, working at 2.5 THz and 4.7 THz, and the low results indicate that the pixel could be at the resolution limit of SOFIA and could be even closer if needed [72]. Liu et al. simulated the self and mutual impedances of a twin-slot-antenna-coupled mixer array at 0.65 THz, which is attached to the back side of a silicon lens array. The relative bandwidth of the slot antenna is simulated to be 13.8%, and the crosstalk of neighboring antennas is very small if the separation is greater than 0.32 λ_0_ [73].

To give a more systematic view of the trade-offs in these different coupling architectures, we summarize the key characteristics in Table 2. The large-format array development comparison revolves around six metrics. These are optical coupling efficiency, filling factor, uniformity, alignment tolerance, crosstalk susceptibility, and fabrication complexity. The assessment incorporates quantitative data from the literature where available. The comparison reveals a shortcoming of traditional methods: there are no quasi-optical architectures currently available that provide both a high filling factor and a uniform array illumination. The fly-eye lens array gives excellent uniformity but its pixel density is limited by the size of each lens element. The single-lens focal plane approach allows a tighter packing of pixels but at the cost of severely distorted off-axis beams.

## 4. A Metasurface Beam Shaper for Planar HEB Mixer Arrays

### 4.1. Metasurface Beam Shaper

**Figure 3:**
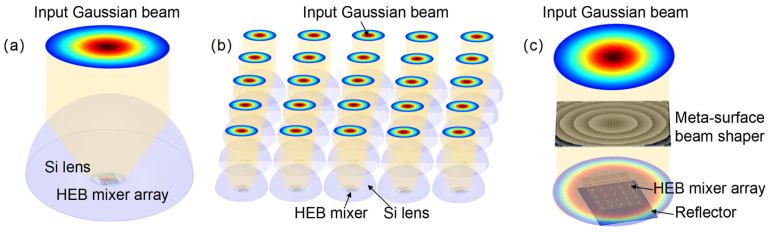
Different approaches for coupling a Gaussian beam to the HEB mixer array. (**a**) Using a large silicon lens; (**b**) using a silicon lens array; (**c**) using a metasurface Gaussian beam shaper.

We propose a coupling scheme for HEB mixer arrays that uses a metasurface beam shaper in conjunction with a planar-antenna array. The metasurface beam shaper is designed primarily for local oscillator (LO) distribution, transforming a single Gaussian LO beam into a flattop profile to provide uniform pump power across the array. As discussed in Section 3, current mixer arrays using a single large silicon lens or a silicon lens array (Figure 3a,b) suffer from either low filling factor or non-uniform illumination. We propose a metasurface beam shaper that transforms a Gaussian beam into a flattop beam at the focal plane, as presented in Figure 3c. Since the metasurface is a lithographically fabricated planar optical element, the array filling factor is determined solely by the antenna spacing on the HEB chip and not the size of individual focusing elements. The flattop illumination allows the pixels to be packed more densely across the HEB mixer array. Additionally, lithographic fabrication allows for monolithic integration with the antenna array, thereby eliminating the mechanical alignment of individual pixels. Since the metasurface beam shaper is a thin optical element, a reflector placed at a quarter-wavelength distance behind the mixer array is used to reinforce the radiation.

### 4.2. Phase Retrieval of the Metasurface Beam Shaper

Beam shaping systems are widely used in the field of laser processing [74,75], lithography [76], laser imaging and detection systems [77], and optical trapping and manipulation [78]. In these systems, a Gaussian beam is shaped into a flattop beam, which offers several advantages, such as energy uniformity and precision. There are different techniques to achieve beam shaping, including the system with aspheric lenses [79], the system with a combination of a fiber bundle and a prism duct [78], refractive optical elements [77], diffractive optical elements (DOE) [80], and subwavelength dielectric metasurfaces [81]. The former two methods use two elements to achieve a flattop beam, which requires fine alignment and are not easy to miniaturize. The latter three methods only require one element, with high energy transfer efficiency and ease of fabrication and miniaturization. Elements based on metasurfaces are designed using subwavelength dielectric structures, whose distribution is meticulously calculated to achieve a precise phase control of the incoming beam. Subwavelength dielectric metasurfaces offer unparalleled design flexibility for achieving complex optical transformations, and we design a metasurface beam shaper to transfer a Gaussian beam to a flattop beam. We selected 0.6 THz and 1.6 THz as the working frequencies, and the same design procedure can be extended to higher frequencies.

To obtain an effective metasurface beam shaper, we need to calculate the phase distribution. There are various calculation methods to obtain the phase distribution, including the geometrical transformation technique [82], analytical beam shaping [83], and the Gerchberg–Saxton (GS) algorithm and its modifications [84,85]. Geometrical transformation is a mathematical coordinate mapping which redistributes optical energy from the input beam profile to a desired output profile based on energy conservation. An analytical solution is a two-step design approach where first a geometrical transformation is derived and then a phase profile of an optical element is calculated to realize it. The GS algorithm was proposed by Gerchberg and Saxton in 1972 to provide a phase retrieval technique for electron microscopy. It is one of the most popular techniques used for designing beam shaping phase masks. The algorithm passes the optical field iteratively between the input plane and the output plane and imposes the amplitude constraint during propagation. The process is continued until convergence. In the terahertz regime, the GS algorithm and its variants are widely used for Gaussian-to-flattop beam conversion using metasurfaces and diffractive optical elements.

In recent years, there has been rapid development in this area. According to Li et al. (2022), a tri-layer metasurface lens was designed to convert a Gaussian beam to a flattop beam at 275 GHz, where the GS algorithm [86] was used to obtain the required phase profile. Subsequently, the same group reported a single-layer metasurface for near-field beam conversion at the same frequency. As a modification to the GS algorithm, the Kirchhoff–Fresnel diffraction equation was employed to account for near-field propagation effects. An experimental conversion efficiency of 80% was achieved [87].

Several modified GS algorithms have been developed to address the limitations of the classical method, including speckle-reduced GS with spherical initial phases [88], complex-amplitude constraint GS for metasurface doublets with transmittance higher than 86% [89], and hybrid GS–genetic algorithms achieving uniformities above 94% [90]. Recent terahertz demonstrations have realized simultaneous beam shaping and polarization conversion with an RMS error of 0.143 [91] and triple-polarization-channel multiplexed flattop beams with over 70% efficiency [92]. In the present work, we adopt the classical GS algorithm for its simplicity and reliable convergence, while acknowledging that future refinements could incorporate these algorithmic advances.

**Figure 4:**
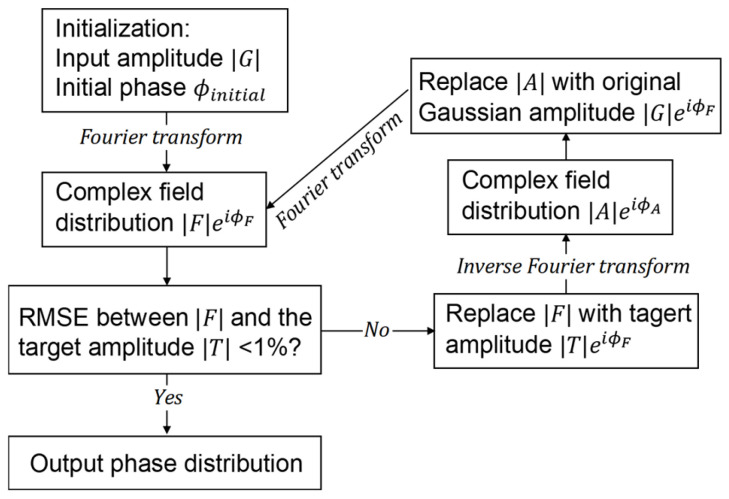
Flowchart of the Gerchberg–Saxton (GS) phase retrieval algorithm. Starting from an initial phase and the known input amplitude, the scheme iterates between the input (metasurface beam shaper) plane and the output (target) plane by alternating Fourier transforms, amplitude substitutions with the target profile, and inverse transforms until the output amplitude converges to the desired distribution (RMSE < 1%). The final phase distribution is then used as the designed DOE profile.

Figure 4 illustrates the computational workflow of the GS algorithm. The process begins with an initial phase distribution $\phi_{initial}$ in the optical element plane and the known amplitude profile |G| of the input Gaussian beam. A Fourier transform of the complex field |G|$e^{i\phi_{initial}}$ yields the output-plane field $|F|e^{i\phi_{F}}$. The amplitude |F| is then replaced by the desired flattop target |T|, while the phase $\phi_{F}$ is retained. An inverse Fourier transform is subsequently applied, producing the updated field $|A|e^{i\phi_{A}}$ back in the element plane. Here, the amplitude |A| is replaced again with the original input amplitude |G|, keeping the phase $\phi_{A}$ unchanged. This iterative loop continues until the root mean square error (RMSE) between the reconstructed amplitude |F| and the target amplitude |T| falls below 1%, at which point the final phase distribution is obtained.

The GS phase retrieval procedure utilized in this work is described as follows. We take the input field to be a one-dimensional Gaussian beam of waist radius $w_{0}$:

(1) $$G(x_{1})=\exp\left(-\frac{x_{1}^{2}}{w_{0}^{2}}\right)\;,$$

where $\;x_{1}\;$ denotes the coordinate in the metasurface plane. The target amplitude profile in the focal plane is defined by a super-Gaussian function of order *P* to approximate a flattop distribution while maintaining numerical smoothness:

(2) $$T(x_{2})=\exp\;[-{\left(\frac{\left|x_{2}\right|}{W/2}\right)}^{P}],$$

where *W* is the desired flattop width and $x_{2}$ is the coordinate in the output plane. In this work we set *P* = 8 to balance edge steepness and convergence stability.

The initial phase guess for the GS iteration is taken as the thin-lens phase profile that would focus a collimated beam to the design distance:

(3) $$\phi_{initial}\left(x_{1}\right)=mod(-\frac{k}{2f_{guess}}x_{1}^{2},2\pi ),$$

where k=2π/λ is the wavenumber and $f_{guess}$ is an equivalent focal length estimated from the numerical grid parameters as $f_{guess}=L_{1}L_{2}/(N\lambda )$, with $L_{1}$ and $L_{2}$ being the computational window sizes in the input and output planes, respectively, and with *N* being the number of sampling points. The modulo operation in Equation (3) reflects the fact that the phase shift imparted by a metasurface is physically equivalent modulo 2π. By wrapping the ideal quadratic phase into the [0, 2π] interval, we obtain a phase profile that can be realized by a finite set of subwavelength scatterers, each providing a local phase delay within the same 2π range.

We model the propagation between the two planes using the Fraunhofer diffraction integral, which is implemented numerically by the Fast Fourier Transform (FFT). For a one-dimensional field $U_{1}\left(x_{1}\right)$, the propagated field $U_{2}\left(x_{2}\right)$ is computed as

(4) $$U_{2}\left(x_{2}\right)=F\left\{U_{1}\left(x_{1}\right)\right\},\;$$

where F denotes the Fourier transform operation. The iterative loop proceeds by alternately imposing the amplitude constraints. In iteration *n*, the forward propagated amplitude |F| is replaced by the target amplitude |T|, while the phase remains the same. After reverse propagation, the amplitude in the input plane is reset to the Gaussian profile |G|. The convergence of the process is checked via the normalized root mean square error (RMSE) between the computed and target amplitude profiles:

(5) $$RMSE=\frac{\sqrt{\frac{1}{N}\sum_{i=1}^{N}{\left(F^{\left(n\right)}\left(x_{2},i\right)-T(x_{2},i)\right)}^{2}}}{{\langle T\rangle }_{ROI}}$$

where the normalization factor is the mean value of the target amplitude over the region where it is non-negligible. The iteration is terminated when the normalized RMSE falls below a preset threshold (typically < 1%), yielding the final phase profile $\phi (x_{2})$ to be realized by the metasurface.

Although the FFT is conventionally associated with Fraunhofer (far-field) diffraction, its use in our GS design is justified by the Fourier-transforming property of a focusing element under the paraxial approximation. The metasurface is designed to impart a phase profile $\phi (x_{1})$ that approximates the ideal thin-lens phase $-kx_{1}^{2}/(2f)$ wrapped into the [0, 2π] interval, which concentrates the incident beam onto the focal plane. According to Fourier optics, the field distribution in the focal plane of a lens is given by the Fourier transform of the field immediately after the lens (multiplied by a quadratic phase factor that does not affect the intensity). Consequently, the FFT operation in our GS iteration corresponds to a physically rigorous description of the focal plane field, not an asymptotic far-field approximation. The finite focal distances of 23 mm at 0.6 THz and 14 mm at 1.6 THz are fully accounted for by the quadratic phase term embedded in the metasurface design. We note that this formulation assumes paraxial optics, which is well satisfied for the moderate numerical apertures (NA < 0.25) in our system. For higher-NA designs, a more general non-paraxial propagator (e.g., the Rayleigh–Sommerfeld integral employed in Ref. [27]) would be more appropriate; this remains an avenue for future refinement.

**Figure 5:**
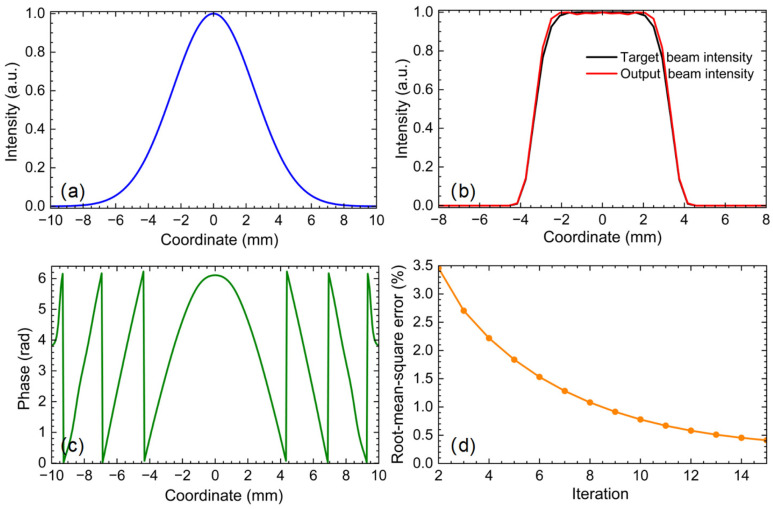
(**a**) Intensity distribution of the input Gaussian beam; (**b**) comparison of the output beam profile (red line) and the target flattop beam profile (black line), showing good agreement; (**c**) the obtained phase distribution; (**d**) normalized root mean square error as a function of iteration number, showing rapid convergence to <0.5% after 15 iterations.

Following the procedure described above, we apply the GS algorithm to a Gaussian beam with a 5 mm waist radius (Equation (1)) and a super-Gaussian target profile of order P = 8 (Equation (2)). The initial phase guess is taken as the thin-lens profile of Equation (3). The GS iteration, implemented in MATLAB, converges rapidly: after 13 iterations the normalized RMSE defined in Equation (5) falls below 0.5%. The resulting phase distribution and the corresponding output intensity profile are shown in Figure 5, together with the input Gaussian and target flattop beams for comparison. The computed output beam profile agrees well with the target, and the flattop region has a width of approximately 4 mm.

### 4.3. Simulation of the Metasurface Beam Shaper

Now that we have the phase distribution, the next step is to design a metasurface to realize this phase profile for light modulation. Many types of metasurface structures have been reported in the terahertz region, which have different characteristics and trade-offs. Phase modulation can be obtained with metasurfaces consisting of gradient C-shaped metallic split-ring resonators. However, they are polarization-sensitive and suffer from ohmic loss [24]. Gradient-index (GRIN) lenses etched in silicon achieve broadband, low-loss performance by varying the effective refractive index through subwavelength holes. Such lenses have been implemented successfully at 90–180 GHz [26]. At supra-THz frequencies (>1 THz), however, the required feature sizes for hole-based GRIN lenses become so small that reliable deep reactive-ion etching becomes challenging, constraining the scalability of the concept to higher frequencies. Tunable metasurfaces based on functional materials like graphene and liquid crystals can enable dynamic reconfigurability suitable for applications like beam steering and active wavefront control [93,94]. However, their complex biasing requirements, limited phase modulation depth, and lower transmission efficiency currently limit their suitability for cryogenic, low-noise HEB mixer arrays.

In contrast, all-dielectric metasurfaces based on high-resistivity silicon pillars offer a compelling combination of advantages for THz beam shaping in HEB mixer arrays [95]. They provide high transmission efficiency (>70% across the required phase range), are polarization-insensitive when using circularly symmetric pillars, and leverage the mature, low-loss properties of high-resistivity silicon at cryogenic temperatures. Although the unit-cell parameters (period, height, and width range) must be re-optimized for each specific frequency, the same silicon pillar design methodology can be readily applied across the THz spectrum.

Critically, the feasibility of silicon pillar-based metalenses for coupling THz radiation to HEB mixers has been recently validated experimentally. Ren et al. demonstrated a silicon-based metalens coupled to a superconducting NbN HEB mixer operating at 1.63 THz—the first experimental realization of this integration scheme [27]. This work demonstrates that metalenses can be integrated with HEB mixers for THz detection and offers a path for compact focal plane arrays. It should be noted, however, that the metalens in Ref. [27] performs point focusing for a single HEB pixel, whereas the metasurface designed in the present work aims at beam shaping—transforming a Gaussian beam into a flattop focal plane profile to enable uniform illumination across a multi-pixel array.

**Figure 6:**
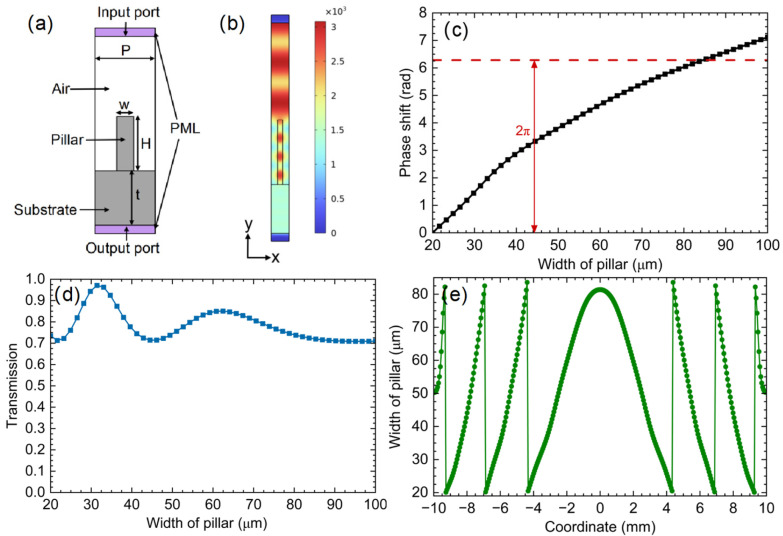
(**a**) A unit cell of the metasurface composed of silicon substrate and silicon cylinder pillar; (**b**) the electric field of the unit cell when the width w of the silicon pillar is 31 μm; (**c**) the achieved phase shift by different silicon pillar widths, covering 2π with w varying from 20 to 100 μm; (**d**) the transmission of the unit cell when the width of the cylinder pillar changes from 20 to 100 μm; (**e**) the arrangement of the silicon cylinder pillar widths to retrieve the phase distribution needed to achieve a flattop beam.

Given the excellent processability of silicon, its proven compatibility with cryogenic HEB operation, and the experimental validation provided by Ren et al., we have chosen the silicon pillar-based metasurface platform for our beam shaper design. Using the RF module in COMSOL Multiphysics, we defined a unit cell of the metasurface, which is composed of a silicon substrate, a silicon pillar, an air box, and two perfectly matched layers (PMLs), as illustrated in Figure 6a. To improve the computation speed and save memory usage, we use a periodic port at the input port and apply periodic conditions on both sides of the unit. PMLs are applied at the top and bottom of the unit cell to absorb waves incident on the boundaries, thereby minimizing reflections. The variables that could be changed to optimize the performance of the unit are the period P of the unit cell, the thickness t of the substrate, the height H, and the width w of the silicon pillar. Through parametric sweeping and optimization of these variables, we derived the optimal distribution to achieve a 2π phase shift and simultaneously high transmission at 0.6 THz, with P = 111 μm, t = 300 μm, H = 400 μm, and w varying from 20 μm to 100 μm. With different values of w, the unit cell could achieve different phase shifts covering 0 to 2π, as shown in Figure 6c. Figure 6b presents the electric field of the unit cell when the width w of the silicon pillar is 31 μm, and Figure 6d presents the transmission of the unit cell when the width w varies from 20 μm to 100 μm, which remains above 70%. The non-monotonic variation in transmission arises from Fabry–Perot resonances within the pillar: as the width changes, the effective modal index varies, altering the round-trip phase and producing transmission maxima and minima. Knowing the relation between the phase and the width of the silicon pillar, we could retrieve the phase distribution we obtained in Figure 5c using different pillar widths, leading to the width distribution in Figure 6e.

The unit-cell simulations were performed with the following additional settings. A linearly polarized plane wave with the electric field oriented along the *x*-axis was incident normally on the structure. The high-resistivity silicon (HRSi, with resistivity >5000 Ω·cm) was modeled with a real refractive index of $n_{r}$ = 3.389, based on reported values for HRSi in the terahertz regime at 4K ambient temperature [96]. The absorption coefficient of high-resistivity silicon in the terahertz range is approximately 0.05 cm^−1^ [97]. At this level, the absorption loss through the 300 μm substrate and 400 μm pillar is well below 1%, and therefore material absorption was neglected in the simulations. The mesh for the model was generated using the physics-controlled mesh option wherein the element size was set to extra fine. The maximum element size in the dielectric region is less than λ/5. A frequency-domain study was conducted, with Floquet periodic boundary conditions on the lateral faces and periodic ports utilized for excitation and S-parameter extraction. The transmission phase reported in Figure 6c is referenced to the phase accumulated through a bare silicon substrate of identical thickness t = 300 μm. The phase shift plotted in this graph corresponds to the delay added by the pillar. Finally, we mention that the unit-cell design assumes the local periodic approximation, ignoring the near-field coupling of neighboring pillars. The limitations of this approximation in regions of high phase gradient are discussed in Section 4.3.

**Figure 7:**
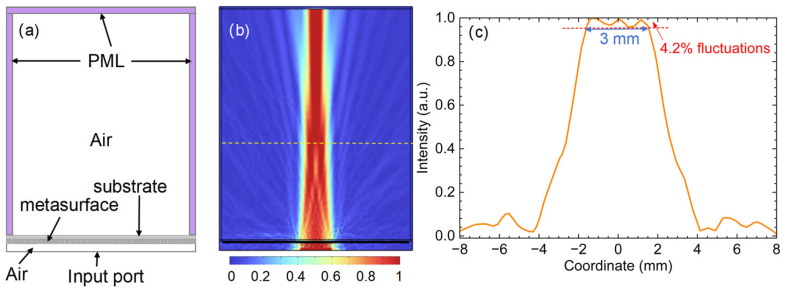
(**a**) The metasurface lens model in COMSOL Multiphysics; (**b**) simulated metasurface beam shaper. The electric field is plotted and the output beam initially transforms into a flattop profile (in the place of the yellow dashed line) and subsequently evolves back into a Gaussian-like beam accompanied by side lobes. (**c**) The output beam profile at a distance of 23 mm from the metasurface. The flat region of the simulated output beam spans approximately 3 mm, with a fluctuation of 4.2% within this region.

The metasurface structure is created in COMSOL according to the width distribution in Figure 6e, and two air boxes are created at the top and bottom of the structure, as shown in Figure 7a. PMLs are placed in the three boundaries of the top air box to absorb waves incident on the boundaries. The boundary in the bottom air box is assigned to be a scattering boundary condition with a defined incident field of a Gaussian beam with a beam waist radius of 5 mm. The simulated electric field distribution is plotted in Figure 7b, and the output beam from the metasurface beam shaper initially transforms into a flattop profile and subsequently evolves back into a Gaussian-like beam accompanied by side lobes. We plot the profile of the output beam 23 mm away from the metasurface structure, indicated with a yellow dashed line in Figure 7b, and the results are shown in Figure 7c. The flat region of the simulated output beam spans approximately 3 mm, with a fluctuation of 4.2% within this region.

The difference between the size of the 3 mm simulated flat region and the size of the 4 mm calculated flat region in Figure 5b could be attributed to several practical departures from the idealized scalar model. The MATLAB calculation treats propagation via Fourier methods under scalar diffraction assumptions, while COMSOL solves the full Maxwell equations, accounting for vector effects, the finite pillar thickness, and non-paraxial behavior. More concretely, the mapping from the continuous GS phase profile to physical pillar widths introduces discretization errors: our unit-cell library sampled only 50 width values between 20 μm and 100 μm, with the remaining phase response obtained by interpolation. In regions where the phase wraps rapidly, this sparse sampling can locally distort the wavefront and erode the flattop extent. A further uncertainty comes from the choice of observation plane. The 23 mm distance was not inherited from the GS procedure; it was located by visually scanning along the axis for the most pronounced flattop profile. A slight axial offset from the true optimal plane alters the apparent width. Finally, the unit-cell design assumes a periodic neighborhood, whereas adjacent pillars in the actual metasurface differ in width and couple via near fields, which is an effect present in COMSOL but absent from the scalar phase map. We note that finite aperture truncation is unlikely to play a role here, since the metasurface spans roughly 40 mm, far larger than the 5 mm beam waist. Taken together, these factors can reasonably account for the observed discrepancy. A rigorous decomposition of the error budget would require systematic parameter studies (denser width sampling, finer axial scans) that lie outside the scope of this proof-of-concept work.

**Figure 8:**
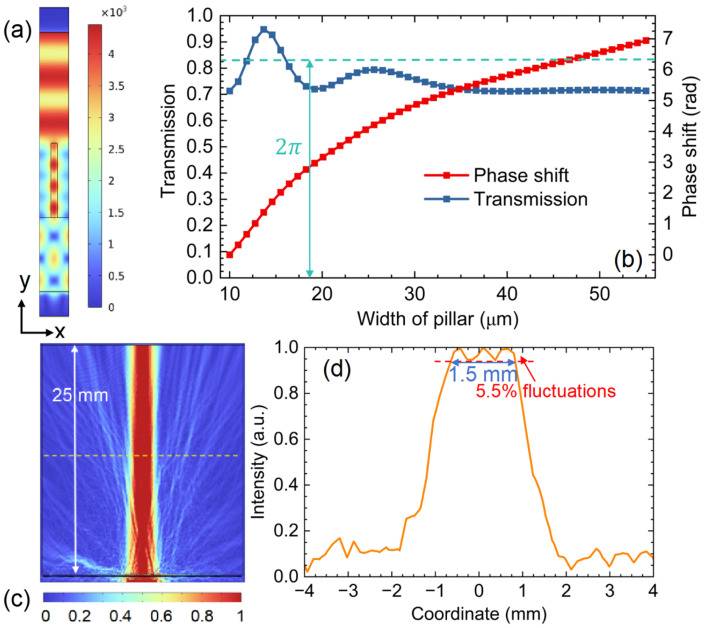
(**a**) Electric field distribution of the unit cell at 1.6 THz with a silicon pillar width of 14 μm; (**b**) phase shift (red line) and transmission (blue line) of the unit cell as a function of pillar width at 1.6 THz; (**c**) simulated electric field distribution of the metasurface beam shaper at 1.6 THz; (**d**) output beam profile at a distance of 14 mm from the metasurface, showing a flat region of approximately 1.5 mm with an intensity variation of 5.5%.

To verify the applicability of this design method in the higher terahertz frequency region, we performed the same calculation by Matlab and simulations by COMSOL Multiphysics at 1.6 THz. Through parametric sweeping and optimization of the period P of the unit cell, the thickness t of the substrate, the height H, and the width w of the silicon pillar, we derived the optimal distribution to achieve a 2π phase shift and simultaneously high transmission, with P = 65 μm, t = 150 μm, H = 150 μm, and w varying from 10 μm to 55 μm. Figure 8a plots the electric field of the unit cell when the width w of the silicon pillar is 14 μm. The phase shift also covers the full 0 to 2π range, and the transmission remains above 70% when the w varies from 10 μm to 55 μm, as shown in Figure 8b.

Based on the optimized unit-cell parameters, we constructed a full metasurface structure in COMSOL following the phase distribution obtained from the GS algorithm at 1.6 THz. The simulated electric field distribution of the metasurface beam shaper is presented in Figure 8c. The output beam profile was extracted at a distance of 14 mm from the metasurface, where the flattop region is formed. As shown in Figure 8d, the flat region spans approximately 1.5 mm with an intensity variation of 5.5%. The 50% reduction in the flat region width compared to the 0.6 THz design is due to the input Gaussian beam waist reducing from 5 mm to 2.5 mm at 1.6 THz. The uniformity remains sufficient for illuminating a compact antenna array. These results confirm that the proposed metasurface beam shaping approach can be extended to higher terahertz frequencies, maintaining good beam uniformity and high transmission.

At 1.6 THz, the optimized pillar dimensions (minimum width 10 μm, height 150 μm) correspond to an aspect ratio of 15:1, which is well within the capabilities of standard silicon deep reactive-ion etching (DRIE). The minimum feature size of 10 μm also remains above the resolution limit of UV photolithography. At frequencies beyond ~2 THz, smaller pillar widths would increase the required aspect ratio and tighten fabrication tolerances, necessitating more advanced lithography or tighter process control. These considerations are important for extending the approach to the multi-terahertz regime.

Since the metasurface beam shaper is designed for coupling LO signals to a HEB mixer array, the intensity variation across the flattop region directly translates to a variation in LO power delivered to individual pixels. To assess whether the simulated intensity fluctuations of 4.2% at 0.6 THz and 5.5% at 1.6 THz are acceptable, we refer to the LO power sensitivity of NbN HEB mixers. As reported in our previous work on LO beam multiplexing [98], the receiver noise temperature of a NbN HEB mixer remains within 10% of its optimal value when the LO power varies by up to ±20% around the nominal pumping level. Consequently, an LO power variation of <6% across the array, which is well within the ±20% margin, indicates that the beam shaping uniformity is sufficient for practical array operation.

To evaluate the bandwidth performance, additional full-wave COMSOL simulations were performed over a ±5% frequency band around both design frequencies. At 0.6 THz, the design-point flattop width is 3.0 mm with an intensity variation of 4.2%, and across the 0.57–0.63 THz band the width remains between 2.2 and 3.2 mm, and the variation stays below 7% at all sampled frequencies. At 1.6 THz, the design-point flattop width is 1.5 mm with an intensity variation of 5.5%. Across the 1.52–1.68 THz band, the width ranges from 0.7 to 1.4 mm, and the variation stays below 10.0%. Since the LO sources for HEB mixer arrays are inherently narrowband and typically operate at a fixed frequency, the main requirement is a single-frequency optimization at the design point. These results confirm that the beam shaping performance is well maintained against small frequency offsets at both 0.6 and 1.6 THz.

## 5. Discussion

This paper reviewed the development of quasi-optically coupled terahertz HEB mixer arrays, with an emphasis on the advantages and limitations of different optical coupling elements and planar-antenna types in array configurations and summarized the noise performance of current planar-antenna-coupled HEB mixer arrays. The conventional “fly-eye” approach based on silicon lens arrays offers good illumination uniformity for individual pixels while suffering from a low filling factor due to the physical dimensions of each lens element. In contrast, the focal plane array with a single lens allows a higher pixel density, but introduces beam distortion and non-uniform illumination for off-axis pixels. This inherent trade-off between filling factor and illumination uniformity complicates scaling HEB mixer arrays to higher pixel counts (e.g., >100 pixels), as required in future astronomical missions.

We propose and numerically validate a metasurface-based beam shaping coupling scheme to address this challenge. A metasurface is designed and simulated to control the phase distribution of an incoming Gaussian beam, thereby generating a flattop beam at the focal plane to achieve uniform illumination of the antenna array. We performed the full design of the metasurface beam shaper at two representative frequencies, 0.6 THz and 1.6 THz, from phase retrieval using the Gerchberg–Saxton algorithm to full-wave simulations in COMSOL. At 0.6 THz, the simulated flattop beam exhibits a flat region of approximately 3 mm with an intensity variation of 4.2%. At 1.6 THz, a flat region of 1.5 mm with an intensity variation of 5.5% is achieved. Compared with conventional lens-array approaches, the proposed scheme based on a metasurface is not constrained by the physical size of individual lenses and can achieve a greater filling factor. The use of flattop illumination eliminates the performance decline connected to off-axis pixels as opposed to single-lens focal plane arrays. Additionally, the all-silicon construction of the metasurface ensures thermal expansion matching with the HEB chip, eliminating differential contraction concerns during cryogenic operation.

It is worth noting that the simulated flat region width at 0.6 THz (3 mm) is slightly narrower than the ideal value obtained from the GS-based design (4 mm). As mentioned in Section 4.3, the difference is due to several practical departures from the idealized scalar design. These issues include the sparse sampling of the unit-cell phase library (50 width values), the empirical determination of the observation plane by visual scanning, and the local periodic approximation that neglects near-field coupling between dissimilar pillars. When considered together with the scalar-to-full-wave propagation transition, these factors can explain the observed ~25% decrease. This discrepancy suggests that for future device designs, the GS algorithm and COMSOL full-wave simulations should be used in combination to obtain a practical metasurface design that accounts for discretization and near-field effects.

A critical aspect that remains to be addressed is the integrated coupling between the metasurface beam shaper and the planar-antenna array. The current work focuses on the design and simulation of the beam shaper itself, assuming ideal illumination of the antenna array. In practical implementation, the interaction between the shaped beam and the antenna array must be carefully evaluated, including coupling efficiency, radiation pattern distortion, and mutual coupling between adjacent antenna elements. We are continuously working towards the following things. First, we will develop electromagnetic models that incorporate the metasurface, free-space propagation, and a planar-antenna array (e.g., twin-slot or spiral antennas) to assess the receiving efficiency and uniformity across the array. In the second step, we will assess the possibility of integrating the metasurface directly onto the HEB chip substrate, which could simplify the optical path and reduce alignment complexity and insertion loss. The next step is experimental validation of a small array (2 × 2, 4 × 4 HEB mixer) to characterize noise temperature, response uniformity, and pixel-to-pixel consistency. Finally, while the input Gaussian beam parameters used in this simulation (5 mm waist at 0.6 THz, 2.5 mm at 1.6 THz) are representative of typical laboratory setups, future work should consider the specific optical characteristics of telescope front-ends, such as feed horns and beam-relay optics, to ensure system-level compatibility.

## 6. Conclusions

This paper has provided a review of quasi-optical coupling techniques for terahertz superconducting HEB mixer arrays, with an emphasis on planar-antenna-coupled configurations. We summarized different optical elements, including silicon lens arrays, metasurface lenses, and metallic mirror arrays; various planar antennas, such as spiral, log-periodic, twin-slot, and annular-slot antennas; and their reported performance metrics in array implementations. The review highlights a fundamental trade-off between filling factor and illumination uniformity that limits the scalability of existing coupling schemes.

To overcome this limitation, we have proposed and numerically demonstrated a beam shaper based on a metasurface. By combining the Gerchberg–Saxton algorithm with simulations in COMSOL, we designed metasurface beam shapers operating at 0.6 THz and 1.6 THz that convert an incident Gaussian beam into a flattop beam at the focal plane. Simulation results show a flat region of approximately 3 mm with an intensity variation of 4.2% at 0.6 THz and a flat region of 1.5 mm with an intensity variation of 5.5% at 1.6 THz. This approach represents a promising initial step toward addressing the trade-off between filling factor and illumination uniformity. Further work, including integrated antenna-array simulations and experimental validation, will be required to fully assess its suitability for large-format HEB mixer arrays.

## Figures and Tables

**Table 1:** Optical element arrays for coupling THz radiation into HEB mixers.

Optical Element	Technology/Material	Fabrication Method	Array (Pixels)	Frequency (THz)	Reference
Lens arrays	Silicon lens array	Direct machining	102; 721	0.44; 0.22	[18,19]
	Silicon lens array	Laser ablation	880; 961	0.35; 0.85	[20,21]
	Silicon lens antenna array	Photolithography and deep reactive etching	5 × 5	0.55	[22]
	Polymer lens array	3D printing	2 × 2	0.34	[23]
Metamaterial-based lens array	Metasurface lens array	C-shape split-ring resonators	5 × 5	0.35–1.05	[24]
Metallic mirror array	Parabolic/spherical mirror	Machining	16	2.5	[25]

**Table 2:** Comparison of quasi-optical coupling architectures for THz HEB mixer arrays.

Metric	Single Large Lens [60]	Fly-Eye Lens Array (GUSTO) [70,71]	Fly-Eye Mirror Array [25]	Fly-Eye Metalens Coupled HEB [27]
Optical Coupling Efficiency	High (on-axis) ✗ Off-axis degraded	High	High	Moderate (early stage) ✓ ~25% demonstrated @ 1.63 THz ✓ Room for optimization
Filling Factor	High (antenna-limited)	Low (lens-diameter limited)	Low (mirror-diameter limited)	Low (metalens-diameter limited)
Uniformity (Pixel-to-Pixel Variation)	Very poor ✗ $T_{rec}^{DSB}$ varies > 100% between adjacent pixels	Excellent ✓ <4% coupling efficiency variation @ 4.7 THz (worst case)	Excellent (expected) ✓ Independent optics per pixel	Excellent (expected) ✓ Independent optics per pixel
Alignment Tolerance	High (single element)	Low (per-pixel alignment required)	Very Low (complex multi-element assembly)	High (lithographically defined, no post-alignment)
Crosstalk Susceptibility	Moderate ✓ >0.32 λ_0_ spacing sufficient [73]	Low ✓ Independent focusing per pixel	Low ✓ Verified for 2 × 2 @ 2.5/4.7 THz [72]	Moderate (to be fully validated)
Fabrication Complexity	Low	Moderate (lens array machining)	Moderate (mirror machining)	High (sub-μm lithography required)

## Data Availability

The raw data supporting the conclusions of this article will be made available by the authors on request.

## Abbreviations

The following abbreviations are used in this manuscript:

DATE5	Dome A Terahertz Explorer (5 m telescope)
DOE	Diffractive optical element
DSB	Double-sideband
GRIN	Gradient-index
GS	Gerchberg–Saxton
GUSTO	Galactic/Extragalactic ULDB Spectroscopic Terahertz Observatory
HEB	Hot electron bolometer
HIFI	Heterodyne Instrument for Far-Infrared
IF	Intermediate frequency
ISM	Interstellar medium
LO	Local oscillator
MgB_2_	Magnesium diboride
MKID	Microwave kinetic inductance detector
MMIC	Monolithic microwave integrated circuit
NbN	Niobium nitride
PML	Perfectly matched layer
RMSE	Root mean square error
SOFIA	Stratospheric Observatory for Infrared Astronomy
TELIS	Terahertz and submillimeter Limb Sounder
THz	Terahertz
